# Laboratory Hyperspectral Image Acquisition System Setup and Validation

**DOI:** 10.3390/s22062159

**Published:** 2022-03-10

**Authors:** Alejandro Morales, Pablo Horstrand, Raúl Guerra, Raquel Leon, Samuel Ortega, María Díaz, José M. Melián, Sebastián López, José F. López, Gustavo M. Callico, Ernestina Martel, Roberto Sarmiento

**Affiliations:** 1 Institute of Applied Microelectronics (IUMA), University of Las Palmas de Gran Canaria, 35003 Las Palmas de Gran Canaria, Spain; amorales@iuma.ulpgc.es (A.M.); phorstrand@iuma.ulpgc.es (P.H.); rguerra@iuma.ulpgc.es (R.G.); slmartin@iuma.ulpgc.es (R.L.); or samuel.ortega@nofima.no (S.O.); mdmartin@iuma.ulpgc.es (M.D.); jmelian@iuma.ulpgc.es (J.M.M.); seblopez@iuma.ulpgc.es (S.L.); gustavo@iuma.ulpgc.es (G.M.C.); emartel@iuma.ulpgc.es (E.M.); roberto@iuma.ulpgc.es (R.S.); 2 Norwegian Institute of Food Fisheries and Aquaculture Research (NOFIMA), 9019 Tromsø, Norway

**Keywords:** hyperspectral images, aberrations, validation, image acquisition

## Abstract

Hyperspectral Imaging (HSI) techniques have demonstrated potential to provide useful information in a broad set of applications in different domains, from precision agriculture to environmental science. A first step in the preparation of the algorithms to be employed outdoors starts at a laboratory level, capturing a high amount of samples to be analysed and processed in order to extract the necessary information about the spectral characteristics of the studied samples in the most precise way. In this article, a custom-made scanning system for hyperspectral image acquisition is described. Commercially available components have been carefully selected in order to be integrated into a flexible infrastructure able to obtain data from any Generic Interface for Cameras (GenICam) compliant devices using the gigabyte Ethernet interface. The entire setup has been tested using the Specim FX hyperspectral series (FX10 and FX17) and a Graphical User Interface (GUI) has been developed in order to control the individual components and visualise data. Morphological analysis, spectral response and optical aberration of these pushbroom-type hyperspectral cameras have been evaluated prior to the validation of the whole system with different plastic samples for which spectral signatures are extracted and compared with well-known spectral libraries.

## 1. Introduction

Over the last few decades, hyperspectral imaging (HSI) technology has gained momentum because of its capability to providing abundant spectral information of the scene, allowing subtle differences between elements that are sometimes imperceptible for other technologies to be uncovered. This has led to its use in remote sensing applications in fields such as as defense [1], security [2] or mineral identification [3] just to name a few, as well as in controlled environments such as in laboratories to conduct experiments and studies of particular materials and products [4,5] or in industrial processes, contributing to the screening of the quality of goods in production [6]. Hyperspectral image processing has been a topic of deep research over the last few decades, as numerous new techniques emerge, from simple spectral index calculations to complex deep learning algorithms, with the purpose of finding a trade-off between results improvements and operations and data simplification [7,8].

Although new developments in acquisition techniques are continuously emerging [9] with improved approaches for capturing both the spatial and the spectral information of the target, for the aforementioned applications in controlled environments, the pushbroom technology remains the most widely used, as it presents a good trade-off between spatial and spectral resolution, with a good radiometric performance that results in datacubes that are spectrally well characterized after the camera has been properly calibrated [10]. Even though the technology has experienced an enormous progress over the last few decades in terms of device miniaturization, electronics and standardization, the acquisition of a meaningful, high-quality datacube is not just a matter of plug-and-play, like with other camera devices. There are a few things to be taken into account when trying to accomplish this task, as it will be discussed in the next lines.

First, the goal is to obtain a three-dimensional (3D) datacube of the sample, containing two-dimensional (2D) spatial information as well as a third dimension of spectral information, using a 2D sensor array. In order to capture all data, it is necessary to perform a sweep capture as it is not possible to obtain all the necessary information in one frame. Pushbroom cameras make use of almost the whole sensor to capture spectral information, so they have great spectral resolution while sacrificing spatial information. In every captured frame, pushbroom cameras capture just one line (1D images) of spatial information (using the other dimension of the sensor to store the spectral information). The ensemble of all of these 1D lines captured together constitutes the regular 2D image. Therefore, with pushbroom cameras, it is required to have a relative linear motion between the camera and the sample to perform a spatial sweep. This can be done by moving either the camera or the sample at a controlled and steady velocity. For the resulting cube to accurately represent the scene and avoid deformations, a proper synchronisation between the applied motion and the acquisition from the camera must take place. This is achieved by developing software packages for both the camera and the motor controlling the linear displacement. Any abrupt acceleration by the motor or vibrations directly impact the data quality.

Secondly, the camera has to be spectrally calibrated, which means identifying each individual wavelength value of the acquired spectral bands. This process is executed on the whole camera system, including both the camera sensor as well as its optical system. On top of that, for each acquisition, an image calibration has to take place, consisting of converting the digital numbers captured by the sensor to the actual percentage of light that is reflected by the objects in the scene. This is accomplished by acquiring two additional frames with the camera, one of a certified material that reflects almost all the light and one with the lens shut. Moreover, for this to work, the scene must be lit with a uniform illuminating source emitting energy at least in the same spectral range the camera is able to acquire.

Last, the camera aberrations caused by the optics and the sensor have to be quantified and corrected in order to be able to use the obtained images in any application, improving its quality and hence, the obtained results.

There exist already commercial solutions that include some of the mentioned points. Some vendors do offer, in addition to their cameras, a setup for the acquisition, including software packages to control the devices [11]. Nonetheless, these are closed solutions that are rather costly and force users to stick to a unique camera vendor and additional features are usually either not an option or substantially increase the overall price. Furthermore, in the scientific literature, some authors have accomplished the task of setting up a pushbroom camera in a laboratory [12,13], but again these solutions are particularized to a specific model, either commercial or self-developed, and are not intended for a more generalized use.

In this work, a system has been developed to acquire images in the laboratory using any Generic Interface for Cameras (GenICam) [14] compliant hyperspectral camera using the gigabit ethernet interface [15]. This interface has been slowly taking over its predecessor, CameraLink, particularly for products for which size and weight are critical. For instance, the Hyperspec-Nano model from HeadWall Photonics (Fitchburg, MA, USA), or some of the models of the Pika series offered by Resonon Inc. (Bozeman, MT, USA) are equipped with such interface. In particular, in this case, tests have been carried out for the FX series from Specim, Spectral Imaging Ltd. (Oulu, Finland), with the FX10 covering the spectral range from 400 nm to 1000 nm and the FX17 covering the spectral range from 900 nm to 1700 nm. Software packages have been programmed to control the camera and the motor separately and a graphical user interface (GUI) has been created to easily interact with the whole system and automatize the entire hyperspectral acquisition.

Additionally, the image calibration process has been automatized, by recording relevant data such as the light source intensity, the sensor temperature or the camera exposure time when acquiring the white and dark references, that let the system select a white reference and dark reference that were previously recorded, so the user does not have to acquire them every time a hyperspectral image capture takes place, simplifying the whole process.

The presented work also introduces a validation procedure for both the camera device and the acquired images. Aberrations such as keystone and smile are very common in pushbroom cameras [16], and usually corrected in a postprocessing phase either within the sensor electronics or afterwards [17]. For instance, the Specim devices used in this work correct these aberrations within the sensor hardware. A testing procedure for evaluating these distortions is presented. Moreover, the spatial morphological deformations that can be present in the acquired image due to the scanning sequence are assessed as well in order to evaluate the accuracy of the synchronisation between the camera and the linear motion. The spectral accuracy of the data is validated using certified material whose signature is well-known in advance.

Finally, a use case is presented where different plastic types are captured with the Specim FX17 camera. The obtained spectra is compared to the state-of-the-art references of the same material to evaluate the spectral quality of the captured data, which constitutes yet another validation method of the proposed system.

Although the presented work has been intended for laboratory use, the hardware and software are perfectly adaptable for industrial applications, with some minor modifications. In typical industrial applications such as quality control, element sorting or classification, making use of vision technologies, the cameras are installed in a fixed position on top and the scanning takes place thanks to a moving conveyor belt where the targets are placed. This perfectly suits pushbroom cameras, so long the conveyor belt speed motion and the camera frame rate are synchronised. It is also very unlikely to have the antireflective coated cage in an industrial installation, so there are some illumination requirements that should be met in order to be able to acquire correct spectral information. As the system would not be isolated in a dark environment, it is crucial to ensure that the dedicated light source is powerful enough to cancel out the effects of the rest of the light sources in the industrial environment.

The developed system is already being used by our group in different applications for research purposes. For instance, as already mentioned, it is being utilised in a plastic detection and classification application for which the spectral information is crucial in order to identify different plastic sorts, as just relying on the colour might be sometimes misleading in this particular scenario. The outcome of such a research could potentially lead to a system able to detect and identify plastics in polluted environments. Another scenario where the system is proving to be valuable is in the analysis of vine species, first aiding in the identification of the particular species and secondly, detecting a plant decease at an early stage.

To sum up, the presented work describes the design and implementation of a custom hyperspectral capturing booth, with a detailed explanation on both the hardware components and the developed software to control the system. Additionally, the system has been validated using different methodologies to test quality and accuracy of both the spectral and the spatial information acquired. These methodologies, which include low-cost and easy to reproduce methods for detecting spectral aberrations such as smile and keystone, are presented and explained. Moreover, the developed system has been used as well in a real-world example capturing different types of plastics in the visual and near infrared (VNIR) ranges, covering from 400 nm to 1700 nm, and the results have been compared with well-known database spectra.

As hyperspectral technologies research is a popular topic with a lot of different applications, we hope that this work can be of help as an introductory manual to any research group that wishes to set up its own hyperspectral laboratory.

The rest of the manuscript is organised as follows. Section 2 describes the proposed acquisition platform setup, first outlining the hardware elements involved in the system in Section 2.1, and secondly, providing a thorough explanation about the software implementations carried out to achieve a proper system operation in Section 2.2. Details about the most relevant hardware components such as the hyperspectral cameras, the motorized linear stage and the illumination system are defined in Section 2.1.1, Section 2.1.2 and Section 2.1.3, respectively. Detailed information about the motor control, the hyperspectral camera control and the user interface application can be found in Section 2.1.1, Section 2.1.2 and Section 2.1.3, respectively. In Section 3, a detailed description of the hyperspectral image acquisition process is provided, first explaining the calibration process and the proposed automation in Section 3.1 and later detailing how the image scan is preformed in Section 3.2. The validation procedure and tests performed to the camera devices are presented in Section 4. First the camera keystone and smile aberrations are measured in Section 4.1. Then the camera spectral calibration is validated using a certified material in Section 4.2. To complete the validation phase, a morphological analysis is performed for the captured images by the proposed system in Section 4.3. In Section 5, a set of different plastic types are scanned with the Specim FX17 camera and the obtained data is analysed, comparing them with hyperspectral signatures available in the literature. Finally, Section 6 discloses the obtained conclusions and outlines further research lines.

## 2. Acquisition Platform Setup

One of the main goals of the designed system is to be able to acquire images with pushbroom hyperspectral cameras in a simple manner, almost right out of the box and with very little or no new adaptations required. The only adjustment needed for each new camera would be a mechanical element that fixes it to the linear stage.

**Figure 1:**
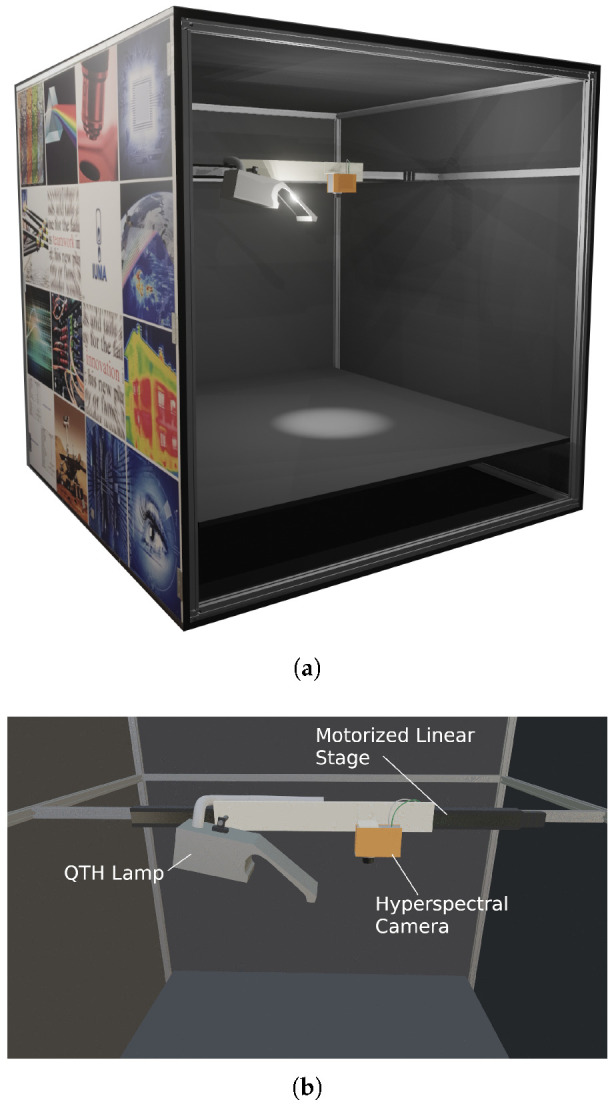
Hyperspectral Laboratory model and detail view. (**a**) 3D model of the acquisition system. (**b**) Detailed view of the main components involved in the acquisition system.

In this section, all the information details of the carried-out design are provided, both in terms of hardware components that make up the whole system as well as software modules programmed to integrate the individual elements. A 3D view of the laboratory acquisition platform system is displayed in Figure 1a.

### 2.1. Hardware Components

The system consists of a hyperspectral camera, a motorized linear stage that enables the linear motion, a light-source-emitting energy uniformly in the range 400 nm to 2500 nm, with intensity regulation controlled by a power supply based on an auto-transformer. The whole acquisition system is contained within a 145 cm-high, 184 cm-wide and 135 cm-deep cage lined with antireflective coating that substantially reduces light leaks and keeps the lighting conditions very similar between two subsequent captures, which is advantageous during the calibration process. A desktop personal computer (PC), model type Mountain with motherboard ASUS Z10PE-D16 WS, placed outside of the capturing booth and Ubuntu 16.04 was used to control the camera and the motor and synchronize both processes during the acquisition. A detailed view of the main components involved in the acquisition system is displayed in Figure 1b.

#### 2.1.1. Hyperspectral Camera

The proposed system is developed to work with pushbroom hyperspectral cameras. This type of camera has a spectral resolution of hundreds of wavelengths which allows an extensive study of the spectral characteristics of the captured targets. However, as this type of camera has a spatial resolution of one line, a spatial scanning is required to capture a 3D hyperspectral cube. Therefore, the camera is mounted in a motorised linear stage that carries out the relative motion between the camera and the target elements.

The main prerequisite for a camera to be compatible with the presented system is that it shall comply with the GenICam standard, which is an interface standard for high-performance industrial cameras. Most recent hyperspectral cameras have adopted this standard as it provides enough bandwidth to convey the huge amount of data intrinsic to the technology and does not need bulky frame grabbers which increase the overall system cost and size.

In this work, two models from the manufacturer Specim have been utilized, the FX10 and FX17, a visual and near infrared (VNIR) and a near infrared (NIR) camera that together cover a spectral range from 400 nm to 1700 nm. Table 1 displays a summary of the most relevant features from both models.

The cameras are mounted one at a time, depending on the spectral range that needs to be acquired. In this case, both cameras incorporate on the sides dovetail salients where a bracket can be fixed. Additionally, a plastic part created with a Ultimaker 3D printer is used to secure the camera to the linear stage.

The connection to the PC is made using a standard ethernet cable with a RJ45 connector plug on the computer side and M12 connector plug on the camera side. The computer network interface card (NIC) shall support a 1000 MBits/s data rate.

#### 2.1.2. Motorized Linear Stage

A motorized linear translation stage is used in this case, to perform a hyperspectral scan with the camera at a constant speed alongside a single axis as well as to position it accurately at a given distance. This accuracy is achieved thanks to a motor microstep size of 0.4961 μm. It typically includes a moving part and a stationary base joined by a bearing system. The position and movement speed are controlled electronically with the use of a motion controller.

In order to produce relative motion between the observed sample and the camera there are two options: either moving the camera and keeping the sample fixed or vice-versa. Here, we have opted for the first, as it usually allows a longer motion range, hence a larger area can be scanned at a single acquisition, obtaining greater output images. For that purpose, the motorized linear stage has to be attached to a wall or a structure in order to have a distance between the camera and the sample. The model A-LST1000B from the manufacturer Zaber [18] has been selected with a travel range of one meter. Table 2 display its most relevant characteristics.

The selected model is able to withstand a 100 kg centred load and 3000 N·cm cantilever momentum, which provides sufficient margin for attaching two hyperspectral cameras and performing an acquisition with a much larger spectral range in one single scan. This is not part of the work that will be covered in this manuscript but is being explored by the group as a future line of research.

The system is connected to the desktop PC using an RS-232 to USB adaptor and controlled using the Zaber Binary protocol described in [18].

#### 2.1.3. Illumination System

The illumination system used in this work is based on a 150 W Quartz Tungsten-Halogen (QTH) lamp with a broadband emission between 400 nm to 2500 nm (VIS and NIR spectral range).

The bulb is installed within a metal casing which has a mirror flap that acts as a paraboloidal reflector, collimating the light beams out of the bulb into a single line transversal to the motion, where the camera is looking at. In between the camera and the reflector, a diffuser has been fixed in order to homogeneously distribute the light along the line and avoid bright spots.

The light source where the lamp is installed is a Techniquip Model 21 DC [19] connected to a fiber optic that transmits the light to the cold emitter or bulb. Using this cold light system has the advantage of considerably reducing the heat transmission to the object being examined and, therefore, the stress that the energy irradiation could cause to it, as opposed to other direct sources of light.

### 2.2. Software Development

The previous section covered in detail the hardware elements involved in the proposed acquisition system. The next step in the integration process should take care of coordinating all these elements together in order to correctly capture a hyperspectral cube. This coordination is carried out with a set of software modules whose implementations are described in this section.

The developed software can be divided in the following applications:

- Motor control module;
- Camera control module;
- User Interface (UI) application.

The first module consists of a set of basic functions to control the motor in charge of moving the hyperspectral camera along a single axis. This module has been written in Python programming language and it makes use of the Pyserial library [20], which simplifies the interaction with the serial port of the machine and enables the communication with the linear stage using a specific protocol. As with most devices commercialized by Zaber Technologies, the communication with the linear stage is done using the RS232 [21] communication protocol at 9600 baud. An accurate control of the motor speed and position is crucial to accomplish a correct hyperspectral datacube acquisition as the scanning result is directly impacted by the precision and the smoothness of the camera motion.

The second module is in charge of controlling the hyperspectral camera device and it has been programmed in C++ language. It is based on the Aravis library [22], which is a glib/gobject-based library for video acquisition using GenICam cameras, implementing the GigEVision or USB3 protocols. This second module manages the camera streaming functionality, the frame acquisition, and all the different parameters of the camera that need to be taken into account to correctly acquire the data.

Finally, the third module is a UI application that has been developed to allow an easy interaction with the whole system. This application is programmed in Python and makes use of the DearPyGui library [23], a fast and powerful graphical user interface (GUI) toolkit for Python. This third module is designed to combine all the functionality available in the acquisition system along with all the possible configurable options in the most intuitive and easy-to-use way. With this application, it is possible not only to graphically configure all camera parameters, set the motor in motion and carry out the hyperspectral captures, but it also aims at giving the user feedback about the system status and provides extra functionality to inspect the captured data after its acquisition.

#### 2.2.1. Motor Control Module

The motor control module is designed to be completely independent and with a simple but robust interface. As already mentioned, the Zaber A-LST Motor Series are controlled via the serial port, supporting both an ASCII as well as a Binary Protocol. In this case, the latter has been used. It consists of simple 6-byte-long messages containing the receptor device ID, the command code which represents the instruction to be executed by the motor, and the data to send. The data take the remaining 4 bytes, the first byte (number 3 in the whole message structure) corresponds to the less significant byte and the last (number 6 in the whole message structure) corresponds to the most significant byte. The bytes have to be transmitted with less than 10 ms delay between each other in order for the motor to correctly interpret the message.

For every outgoing message to the motor there is always a feedback message which has the same structure as the outgoing message. While the device and the command match in both incoming and outgoing messages, the data can be different depending on the instruction. For example, in a *Return Current Position* command the data in the outgoing message is ignored and in the incoming message it contains the current position value.

Messages from the motor are returned as soon as the action has been finished. This means that while messages could be sent immediately back, others can take seconds or even minutes to arrive. For this reason, both sending and receiving actions are executed in separate threads, one for the outgoing and one for the incoming messages, in order to avoid stalling the main thread, which keeps track of all pending actions in a list. This allows a thorough implementation of all motor actions which include interrupting the movement with a new position to move, performing an emergency stop, or even asking the motor for position feedback during the movement action. In fact, to allow the UI application to precisely show the current motor position at every time, this module automatically runs the *Return Current Position* command periodically when the motor is in motion. This way, the user can always have an updated and precise information about the motor current position. The multithreading nature of the package avoids any kind of serial port multiple access problems and concurrences, which is critical, as different commands are being sent to the motor and incoming responses are being received asynchronously.

#### 2.2.2. Camera Control Module

One of the starting premises of the developed work was to be able to control hyperspectral cameras that complied with the GigEVision [24] standard. In a previous work [25], this task has been achieved by using a third party software, called eBUS SDK from Pleora. Nonetheless, this has proven to work in some particular scenarios where specific hardware platforms were used, but not in all situations. Additionally, being a closed solution, it is rather complex to expand the standard functionality with specific project requirements as the code is not accessible.

For all these reasons, we have chosen to look for an open -ource solution, which has proven to fulfil all the project requirements. It has been successfully tested in different hardware development kits, NVIDIA Jetson Nano or Odroid XU4 among others, and it can handle the data bandwidth seamlessly.

On the downside, the Aravis project [22] does only compile in Linux as well as in MacOS environments, but this is not an issue as all the hardware platforms we intend to run the code in are Linux based.

As mentioned earlier, the module has been programmed in C++, but in order to maintain consistency with the rest of the modules, a Python wrapper for the camera control module has been created, supported by the Pybind 11 library [26]. The wrapper provides an interface to the functions developed within the module.

The camera control application let the user manage the main camera features and actions such as starting and stopping the camera streaming, acquiring captured frames, writing those frames to disk, modifying any camera capturing parameter such as exposure time or frame rate.

#### 2.2.3. User Interface Application

A GUI has been developed to integrate everything together in a software that is easy to interact with by a user. The program has a main bar with four elements, *Settings, Theme, Tools* and *About* that are used for a general software configuration, the rest of the display area is reserved for the interaction with the acquisition system, organized in four different tabs, that are described next.

**Figure 2:**
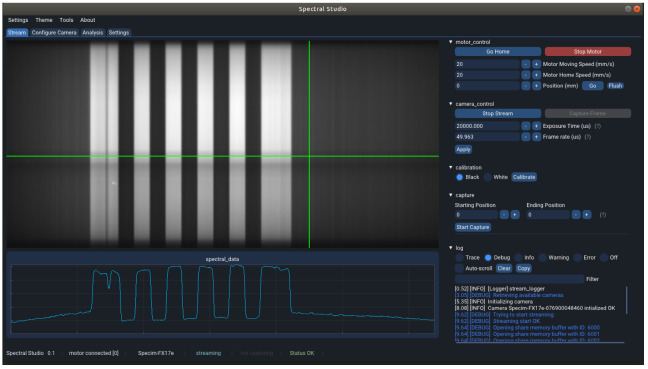
Stream tab of the application user interface.

The main tab, called *Stream*, is the one used to capture the hyperspectral data. Figure 2 shows a snapshot of the tool, streaming a chessboard pattern, which can be seen on the top left-hand side where a visualization widget that shows the last captured buffer from the camera when streaming has been laid out. The data shown in this widget corresponds to the captured frames in x-lambda format, this means that in the *x*-axis of the widget the spatial pixels are represented and in the *y*-axis the spectral information corresponding to each of the mentioned pixels is shown. The green cursors are hovered over the image to select a row and a column within the frame to be displayed in the plot underneath. In Figure 2, the spatial pixels curve, corresponding to the horizontal line, is being disclosed which let us see how well the camera is focused by checking the steepness of the lines. In the *Settings* tab, the widget can be switched to plot the spectral dimension, which corresponds to the vertical line.

On the right hand side, the controls to interact with the other modules have been laid out. First, on the top, the motor controls let the user initialize the connection with the motor, change the speed, set the motor in motion and move it to a specific position and stop it at any time. Right below, some basic camera controls are displayed, that allow setting the exposure time and the frame rate of the camera acquisition and start and stop the stream.

Next, the controls to perform a calibration and a hyperspectral acquisition scan are available. These open popups for a better interaction with the user, which will be further detailed in Section 3.

At the bottom right-hand side, a logging window shows some tool feedback for the user to be informed whether the modules and the software are working properly. For instance, if the camera has successfully initialized or the data streaming is taking place properly.

Both the camera controls as well as the capture/calibration controls are faded out by default, and are only enabled once the application has connected to a camera. This is done in the second tab, *Configure Camera*. A list of all available GenICam complying cameras found in the system is displayed and the user is allowed to pick one to connect to. Once the program has connected to the camera correctly, some controls are enabled to modify different camera parameters, including the ones available in the first tab, the exposure time and the frame rate. If the camera is not performing any capture and is not streaming either, this tab allows the user to disconnect the camera.

The third tab, *Analysis*, let the user import any hyperspectral image captured with the tool and graphically analyse the data. With a functionality similar to the one displayed in Figure 2, using the green cursors again, the user can select several pixels and display their spectral signatures in a plot underneath. This allows a first inspection of the captured data providing a very valuable overview that leads to some further actions. Repeat the acquisition if something went wrong or requires special attention or move on with other captures.

Finally, the fourth tab, *Settings*, adds additional controls to set up the application performance and behaviour.

## 3. Hyperspectral Image Acquisition

The acquisition of a hyperspectral datacube with a pushbroom camera is not as straightforward as it is for a standard imaging device. There are several system variables that have a great impact on the resulting image: the light source intensity, the motor speed or the working distance (the distance from the camera to the sample), to name a few, have to be considered when configuring the acquisition parameters in order to obtain high-quality data.

For the purpose of accomplishing the aforementioned task, the developed application contains the functionality to assist the user as much as possible to obtain the best possible outcome. Section 3.1 details how the calibration methodology is carried out and simplified by the application, whereas Section 3.2 explains how the actual hyperspectral acquisition is performed and the different capturing parameters are set up.

### 3.1. Calibration Methodology

The raw frames captured by the hyperspectral camera are a measurement of the sensed energy per sensor pixel, each pixel value expanding from 0 to the value defined by the camera pixel depth. However, the sensor response is not uniform across the covered spectral range. The consequence is that, even if the same amount of radiance hits equally every sensor pixel, the digital value measured may differ, especially for different wavelengths. Additionally, the illumination conditions may not be uniform across the covered spectral range. These facts make not possible to directly use the raw images, which are affected by the sensor response, for the subsequent hyperspectral imaging applications.

In order to solve the aforementioned issue, the captured images are converted to reflectance values, in such a way that each image value is scaled between 0 and 1, representing the percentage of incident radiation that the scanned object reflects at each specific wavelength. The procedure is explained with Equation (1), which shows how the raw data is calibrated for obtaining its corresponding reflectance values:

(1) $$\mathrm{reflectance}=\frac{sensed\_bitarray-dark\_reference}{white\_reference-dark\_reference}$$

In Equation (1), *sensed_bitarray* represents the raw data values of the scene captured by the camera, and *white_reference* is an image of a Zenith Polymer white calibration panel which is certified to reflect more than 99% of the incident radiation in the spectral range covering from 400 nm to 2500 nm. The certified spectral signature of the white reference material (Spectralon Diffuse Reflectance Material, Labsphere) is used to precisely calibrate all captured images by using the corresponding reflectance value for each individual wavelength. It is placed below the camera so the white reference panel occupies the whole camera sensor. Up to 200 samples at the exact same exposure time as the one used during the acquisition of the raw data are taken and then averaged. Those repeated frames captured with the exact same conditions have been also useful for calculating the repeatability of the capturing system (i.e., the light source and the hyperspectral camera). The obtained mean standard deviations for both the FX10 and the FX17 cameras are 0.11% and 0.05%, respectively. Finally, *dark_reference* represents the minimum values that the sensor measures when no radiance is hitting it. In order to obtain the dark reference, the camera lens is completely closed and again, 200 samples are taken and then averaged.

A very common practice when acquiring hyperspectral data in the laboratory is to obtain the white and dark references prior to every image scan. While this process is rather quick, it can be tedious to constantly repeat it before every capture. One of the main advantages of having the system enclosed in a cage coated with antireflective material is that just a few parameters have a direct impact onto the white and dark reference data. These parameters can be stored in a separate file along with the reference so every time an acquisition is taking place at the same or at very similar conditions, these stored references can be used for the calibration instead.

Consequently, the application saves a Javascript object notation (JSON) file along with every dark and white reference, containing the following information: the hyperspectral camera model, the light source knob position which is a direct indication of the power outcome and light intensity (only relevant for the white reference), the camera exposure time and the camera sensor temperature, as these parameters are the ones that most affect the capturing output. The sensor temperature has an impact in the amount of noise present in the output image, which increases with the raise of sensor temperature. While some hyperspectral cameras provide a sensor cooling system, this is not the case for the cameras used in this work, so the developed system simply reads out the sensor temperature and stores it along with the other relevant information for calibration purposes later on.

**Figure 3:**
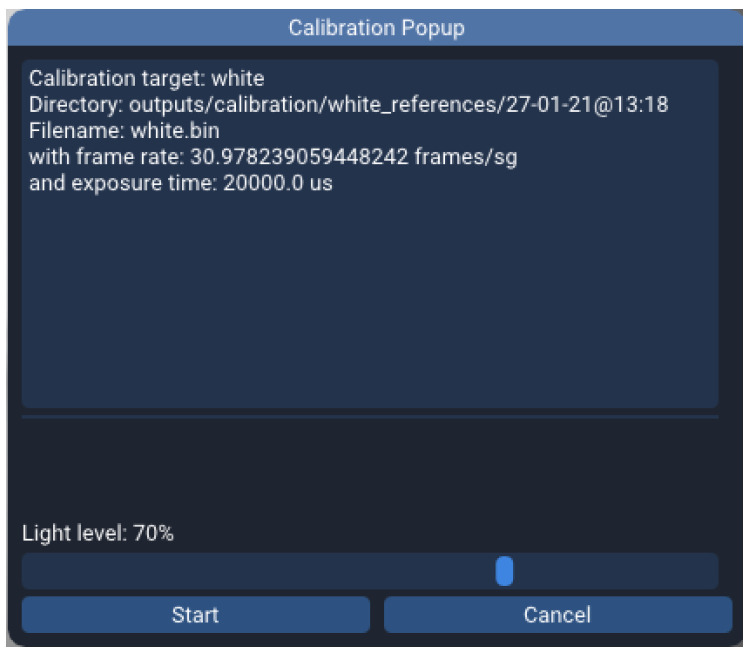
Calibration popup window.

Additionally, the user only needs to manually specify the light source knob position while capturing a white reference, as the rest of the information is available by the system and hence, automatically dumped to the file. Figure 3 shows the calibration window pops up when the user attempts to perform a white or a dark calibration. On top, the data automatically gathered by the application to be saved on the file is displayed, and on the bottom a bar widget where the user selects the mentioned knob position in percentage.

After the user press the start button, the application captures the reference frames and stores them in memory along with the JSON file into a directory that bears the current date and time in its name. This way, when more than one reference is suitable for performing a calibration, the user is prompted to pick one.

### 3.2. Image Scan

In order to perform a hyperspectral image scan, the acquisition geometry has to be taken into account. A few parameters have to be either input by the user or gathered from the camera interface in order to perform the appropriate calculations. These parameters are:

- Working distance, *h*;
- Camera field of view (FOV), α;
- Camera number of spatial pixels, spatial_sampling;
- Camera frame rate, FPS.

The working distance, *h*, is defined as the distance between the camera and the target, and it is manually defined by the user after having measured it with a tape measure. Later, the value is corrected applying a factor as explained in Section 4.3. The FOV is given by the selected optics, in the software each camera model has been assigned a predefined value which is stored in memory but it can also be input by the user should another optical lens be used. This default FOV value is obtained from the camera specifications provided by the manufacturer. The spatial pixel sampling depends on the hyperspectral camera as well as on the selected spatial binning; hence, this value can be retrieved at any time from the interface. The camera frame rate is limited by the selected exposure time which in its turn depends on the light conditions. The exposure time can be fine-tuned using the previsualization widget that prevents a saturated data or a too low signal.

Based on the given parameters, the ground sampling distance (GSD), which represents the size of one pixel on the ground, can be obtained applying the formula defined in (2).

(2) $$\mathrm{GSD}=\frac{2\times h\times \tan\frac{\alpha }{2}}{\mathrm{spatial}\_\mathrm{sampling}}$$

Given the GSD, and the camera frame rate, the speed of the linear stage can be calculated following the expression in (3).

(3) speed=GSD×FPS

**Figure 4:**
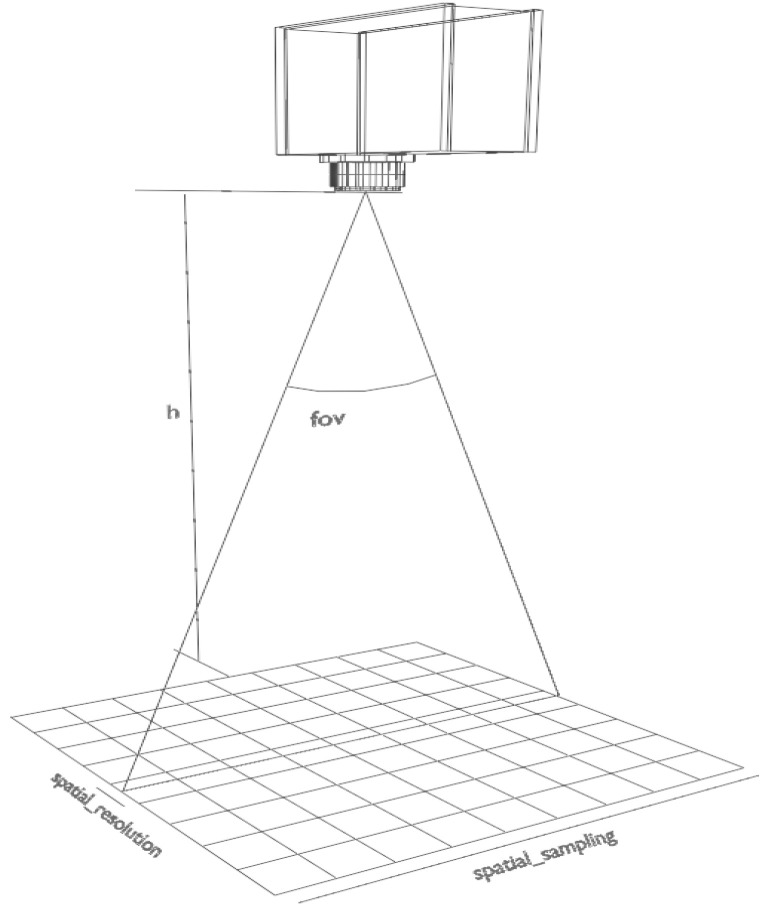
Diagram of the capturing system.

Figure 4 displays a diagram of the geometry involved in the acquisition including the hyperspectral camera and the parameters mentioned earlier.

The units of the GSD and the speed are directly related to the input units of the working distance; for instance, if the distance is defined in centimetres, the GSD units are cm/px and the speed units are cm/s.

**Figure 5:**
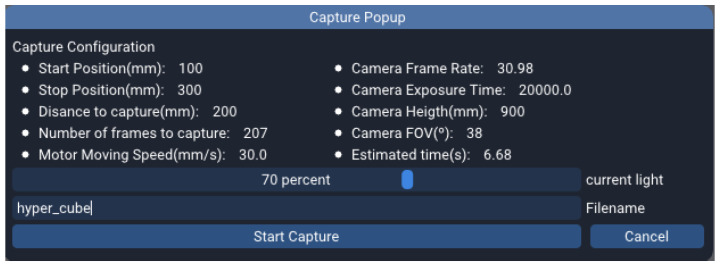
Capture popup window.

The interaction with the GUI happens through the window popup that is displayed in Figure 5. Aside from the parameters mentioned earlier, the user has to define the start and stop positions of the linear stage, in absolute distance measured from the homing position which corresponds to 0 mm. The longest travel distance that can be given is 1000 mm.

The rest of the values are calculated internally by the software, these are: (1) the travel distance, obtained by subtracting the start position from the stop position; (2) the number of frames to capture; (3) the motor speed; and (4) the estimated capturing time. Finally, the light intensity power level is manually input by the user.

Even though most capturing parameters are calculated and set up automatically, a minimal user intervention is required. First, the user has to manually turn on the light source and set its corresponding power level. Then, it is necessary to configure the camera exposure time to ensure the capturing information is not overexposed or too dark. Normally, it is preferable to set the light power to its maximum output value and then adjust the camera exposure time to the maximum value without overexposing the image. This way the acquisition makes the most of the camera pixel depth without losing any information, hence, improving the overall signal-to-noise ratio (SNR).

The image visualisation widget presented in Figure 2 can be used to easily modify the camera exposure time to the optimal value as it provides real time feedback of the captured frames. If the selected exposure time is inconsistent with the current frame rate, the latter is automatically corrected to the minimum valid value.

The aforementioned procedure is applicable for 2D scanning, but it is also perfectly possible to have the camera in a fixed position and capture just one spatial line, in case the spatial features are not required. In this case there would be no need to configure starting and ending scanning positions.

The capture popup window presented in Figure 5 allows the user to review the parameters calculated by the system for the acquisition that is about to take place and give the scan output a filename. The tool will give feedback during the capturing process, as for instance, the motor position or the number of frames already captured. Once the scan is over, the application will automatically look for a suitable black and white reference in the database to perform the calibration. In case no references are found with the adequate characteristics, the user will have the option to manually select a black and white reference from disk, or to dismiss the calibration process. In case more than one suitable references are found, they will be prompted to the user in chronological order so the preferred one can be chosen.

Once the calibration process has ended, the resulting binary file is stored along with the raw binary images, the ENVI [27] header file and a JSON file containing the system configuration used for the capture, inside a unique folder in the application outputs directory. As with the calibration JSON file, the capture JSON file contains a fingerprint of the system status and configuration for the capture. It contains information such as the light intensity level, all the relevant camera parameters, the motor movement speed, etc.

This generated directory can be *sent* to the *Analysis* tab, where the acquired data can be explored. Further details of the options available in this window are explained in Section 5.

## 4. System Validation

Considering the complexity of the processes involved in the hypercube acquisition, it is very important to validate the captured data in order to be able to use them in any real application. For this purpose, in this section a set of tests are defined to asses the performance of the camera itself on the one hand, and the performance of the entire system working altogether on the other.

The aberrations caused by the optics and the sensor and the spectral response are measured on the camera device. Image morphological distortions caused by an uneven synchronisation between the camera and the linear motion are measured on the captured spatial data. In the next lines, the procedures followed in each individual test are explained and presented together with some obtained results.

### 4.1. Aberration Measurements

The complex design of pushbroom hyperspectral cameras bears the risk of optical aberrations in the registered spatio–spectral frames, called keystone and smile [16]. This, combined with pixel nonuniformity distortions caused by an uneven distribution of the incoming light and bad or dead pixels usually found in imaging sensors, drastically decreases the quality of the acquired images unless these effects are corrected.

The keystone effect is a spatial distortion that directly affects the purity of the spectral information of a pixel. Cameras with high keystone influence will mix the spectral information of a point with that of its surroundings, which can negatively affect an hyperspectral application performance. In order to display and measure the keystone effect, we proceed by capturing a frame of a single light point which would produce a line along the spectral dimension of the frame given that the light emits along the whole sensor spectral response. With this line being narrow enough, one can measure how much it deviates from an ideal line due to the keystone effect. One key feature that Specim includes in their hyperspectral cameras is the implementation of hardware-correction algorithms which they call Automatic Image Enhancement (AIE). This feature allows the cameras to automatically correct the effect of spectral aberrations such as smile or keystone along with other image corrections such as nonuniformity and bad pixel replacement. In addition to this, the AIE corrections can be disabled at any time allowing us to capture both raw images along with AIE-corrected images. This enables a proper comparison of the effects of the AIE correction algorithms applied to both kinds of images (with AIE enabled and disabled), and lets us ensure that the aberration effects are being correctly detected.

**Figure 6:**
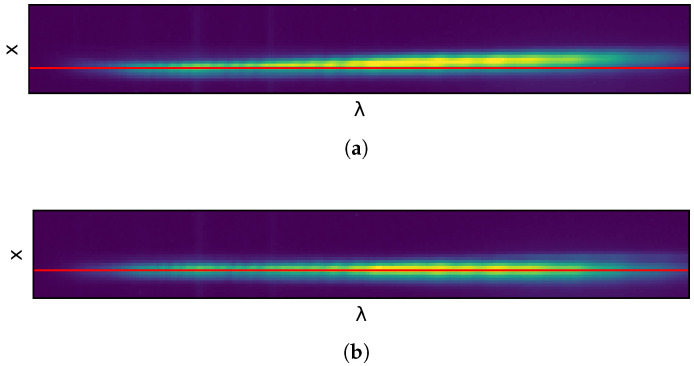
Representation of the keystone effect on individual frames. (**a**) With hardware aberration correction disabled. (**b**) With hardware aberration correction enabled.

Figure 6 shows the mentioned captured frame with the horizontal line along the spectral axis with the hardware corrections off, in Figure 6a and on, in Figure 6b. In order to assist the reader in the visual assessment of the line inclination, in red, a perfectly horizontal line has been added for comparison.

Quantifying the keystone effect for each individual pixel within the captured frame would require a light source that produces a very narrow line so it can be moved and measured with a subpixel precision [28]. While using expensive and specialized equipment is out of the scope in this work, it is possible to obtain a rough numerical value for the keystone at the edges of the sensor where the effect is more pronounced and at the center of the sensor. Table 3 displays the results for both cameras with the AIE corrections enabled and disabled, as well as the improvement measured in percentage obtained by applying the AIE algorithm. These values have been obtained by fitting a straight line to the point light source captured in both frames, with and without AIE correction. The difference in the spatial pixels between the end and beginning of the fitted line provides the keystone measurement. As seen in Table 3, the AIE correction reduces up to a 98.07% of the keystone. The correction factor is calculated using (4).

(4) $$\mathrm{correction}\left(\%\right)=\frac{\left|\left|K_{w/o}-K_{w}\right|\right|}{K_{w/o}}\times 100$$

where, $K_{w}$ refers to the measured keystone value with the AIE correction enabled and $K_{w/o}$ to the measured keystone value with the AIE correction disabled.

**Figure 7:**
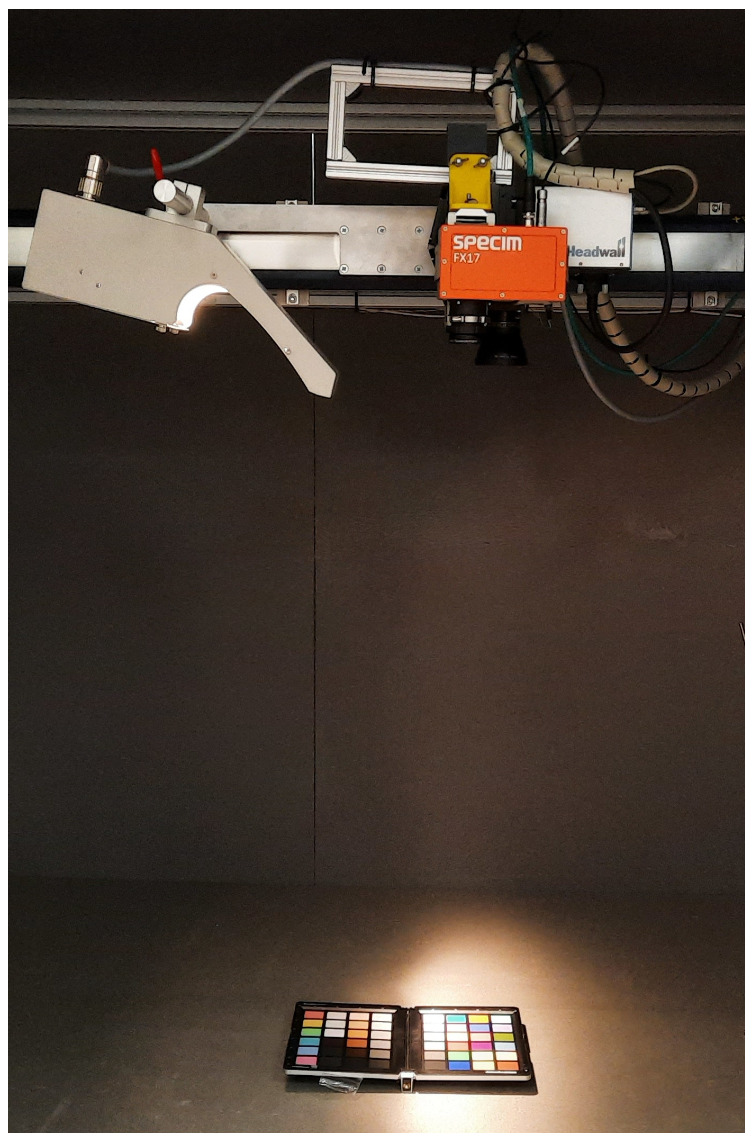
Color checker acquisition.

Measuring the spectral aberrations caused by the smile effect would require a filter between the light source and the camera lens that produces a line along the spatial pixels, perpendicular to the line represented in Figure 6. As, again, this work does not intend to explore the use of specialized equipment, this effect has not been quantified but it has been detected by performing a simple experiment. This consists of capturing an homogeneous target in the center of the camera’s FOV and the same target placed on one of its sides. Due to the smile effect, the spectral response will be shifted when comparing both measured results. In this case a *Datacolor* [29] checker board has been used for the purpose of the analysis. The board is scanned as depicted in Figure 7, showing the laboratory setup when it is placed in the middle of the camera FOV. Another acquisition of the color checker is made placing it on the camera FOV left-hand side. A 10×10 pixels box from the blue square within the color checker is selected for the analysis. The mean value of those 100 pixels is computed as the representative spectral signature of the color.

**Figure 8:**
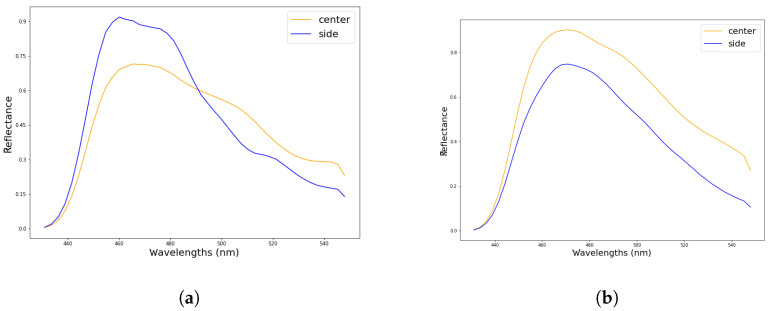
Representation of the smile effect by measuring the same element on different camera FOV positions. (**a**) With hardware aberration correction disabled. (**b**) With hardware aberration correction enabled.

The spectral signature of the blue color captured in the center of the image and on its side can be seen in Figure 8. Figure 8a shows the spectral crossover between signatures that happens when the smile effect is not being corrected with the AIE algortihm. Figure 8b displays both curves parallel to each other in the entire range, after the correction algorithm has been enabled. The reason behind the amplitude differences is due to an uneven light distribution between the center and the side pixels.

The experiment has been conducted with the FX10 camera, as it is able to capture in the visible range. In Figure 8, the spectral range has been cropped for simplicity as the range above 550 nm does not provide any relevant information and the curves remain just parallel to each other.

The results obtained for the proposed experiments prove that the Specim AIE aberration correction algorithm properly corrects both the smile and keystone effects present in pushbroom cameras.

### 4.2. Spectral Response

The third dimension of the acquired hypercube represents the spectral bands. In order to assign a specific wavelength to each individual band, the camera has to be calibrated against a traceable reference device, in a process that is defined as spectral calibration. This enables a data comparison across different platforms.

The response of each individual pixel in the spectral dimension is usually modelled using a Gaussian curve, such as the one represented in Equation (5).

(5) $$f\left(\lambda \right)=k_{0}\;e^{\frac{-{\left(\lambda -\lambda_{0}\right)}^{2}}{2\sigma^{2}}}+b_{0}$$

where $k_{0}$ represents the height of the Gaussian curve, $\lambda_{0}$ is its center, σ its standard deviation and $b_{0}$ is a bias parameter. The idea is to find out the parameters of the Gaussian function, *f*, by using a few discrete measurements and curve fitting techniques, so that later the system response for each individual wavelength, λ, can be determined.

The spectral calibration procedure aims to provide the center wavelength for each channel ($\lambda_{0}$) and the spectral resolution or full-width half-maximum (FWHM), directly dependent on the standard deviation of the Gaussian curve according to the expression: $\mathrm{FWHM}=2\sigma \;\sqrt{2ln2}$, specified in [30].

In order to be able to obtain the mentioned parameters, a monochromator is often used, which produces a beam of a narrow band of wavelength. The method consists of a polychromatic light illuminating the slit of the monochromator that outputs a monochromatic light, which is then collimated to span the entire FOV of the imaging spectrometer. The monochromator shall emit beams in wavelengths stepping intervals smaller than the camera spectral resolution by varying the grating angle and the slit size [30,31]. After the sweep in the entire spectral range of the camera has been performed, a linear fitting process takes place to find out the Gaussian curve parameters for each individual channel.

Camera manufacturers usually own this type of equipment in their labs; hence, they are able to perform the calibration for every device they ship to their customers. In this particular case, for the cameras being tested in this work, the spectral calibration data has been delivered in separated files. In these files, each sensor pixel row is associated to a specific wavelength, which is required by the developed application in order to build the ENVI header of the captured hyperspectral image.

It is important to highlight that the camera slit width has great impact on the Gaussian response of each individual channel: the narrower the slit, the purer the spectral response (narrow Gaussian curve). On the other hand, a narrow slit lets less light through to be captured by the sensor. This translates into the need of a longer exposure time that would reduce the capturing frame rate drastically. Therefore, manufacturers have to find a trade-off between both aspects when designing their devices.

In this Section, the focus is set on validating the given spectral calibration files following the same procedure defined in [32]. The process is accomplished using a calibrated Zenith polymer, whose spectral signature is provided by the manufacturer.

**Figure 9:**
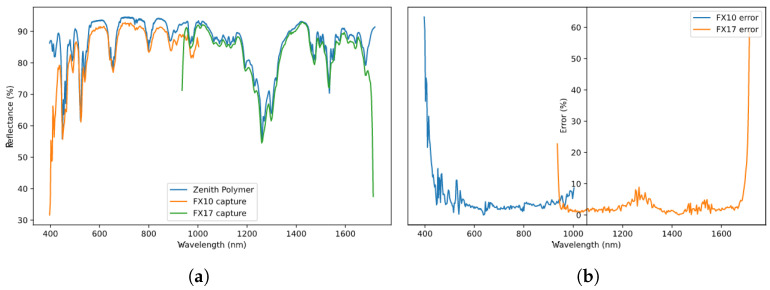
Camera spectral validation using a Zenith Polymer material. (**a**) Polymer signature comparison. (**b**) Signal error.

A hyperspectral image of that polymer is acquired with the proposed system, one for each camera, then cropped, selecting just the pixels corresponding to polymer material, and is finally averaged to obtain a single spectral signature that can be compared to the data given by the polymer manufacturer. Figure 9a displays such a comparison, where both the polymer signature provided by the manufacturer and the signature obtained by both cameras have been overlapped. In Figure 9b, the raw error between the certified signature and the captured signature, calculated as the percentage difference between the reflectance values for every wavelength as shown in Equation (6), is displayed.

(6) $${\mathrm{error}}_{wl}\left(\%\right)=\frac{\left|\left|X_{wl}-R_{wl}\right|\right|}{R_{wl}}\times 100$$

Here, *R* refers to the real (certified) values, *X* to the detected (captured) values and the subindex wl indicates that the operation is performed for each individual wavelength.

On the edges of the sensor, the error is much more pronounced due to the poor signal to noise ratio (SNR) of the camera in that part of the sensor array. For the center wavelengths, the error remains below 10% for each individual band which is considered as acceptable. Should higher error rates appear between both curves, especially in the center wavelengths, the spectral calibration shall then be repeated.

### 4.3. Morphological Analysis

In Section 3.2, the calculation of the ground sampling distance (GSD) was presented based on camera parameters and the distance from the camera to the target object. The accuracy of this value is of key importance in the obtained image as it directly impacts the scanning speed. Errors in measuring the distance from the camera optics to the target and the nonuniformity of the pixel size along the scanning line make the real GSD value differ from the theoretical calculation presented in Equation (2).

**Figure 10:**
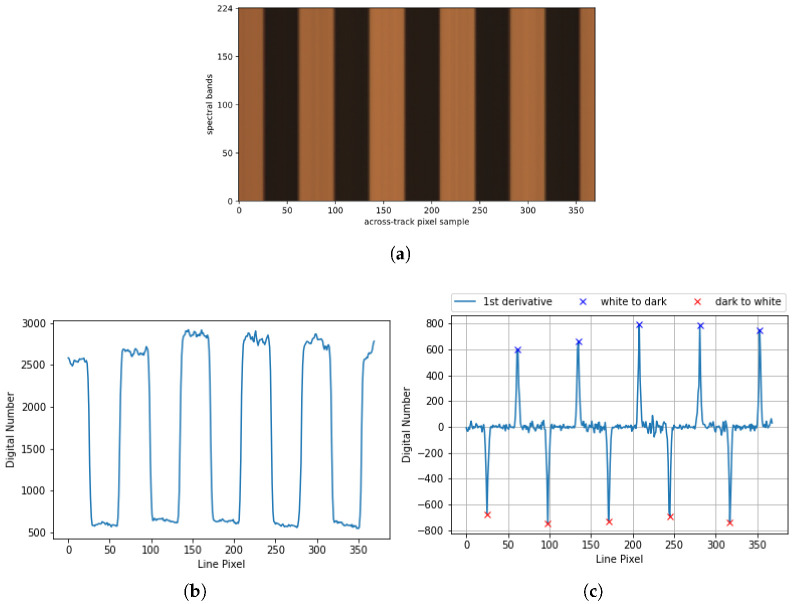
GSD empirical measurement using a chessboard pattern. (**a**) Chessboard pattern x-lambda image. (**b**) The centre line of the frame displayed in (**a**). (**c**) First derivative of plot displayed in (**b**).

For the aforementioned reasons, an empirical measurement of the GSD has been performed using a chessboard pattern with 5 black and 4 white squares per line with a side of 23.3mm per square. Figure 10a shows the x-lambda image of the frame, representing on the *x* axis the spatial pixels and on the lambda axis, the spectral bands. The image has been cropped on the sides in order to just retain the pixels corresponding to the pattern. Figure 10b, shows the plot of the spatial pixels marked at spectral band 112, right in the middle of the frame. As can be seen, the transitions between the white and dark color are represented by an abrupt change in the acquired pixel radiance. In order to better detect these transitions, the first derivative of the plotted data has been calculated and represented in Figure 10c, where the blue cross marks represent the color transition from dark to white and the red cross marks the transition from white to dark.

The distance in pixels between a blue cross and a red cross account for the length of a square in pixels. Dividing the square size in mm, by this amount, provides the GSD value, as it represents how much spatial data is being represented in each pixel. In Table 4, the empirical and theoretical GSD values are being displayed. The first one is obtained as the quotient between the measured square size in mm and the average of all dark and white square distances in pixels. The test has been repeated placing the pattern on the camera center, on the left and on the right hand sides.

The theoretical value displayed in Table 4 has been calculated using Equation (2) where the distance from the camera fore-optics, *h*, has been measured with a meter band, giving the value 920 mm. The error shown in the table between the theoretical and the empirical value have been calculated as the percentage absolute difference between both values using Equation (7), where, Th refers to the theoretical value and Emp to the empirical (calculated) value. The deviation between the empirical and theoretical values increases as the distance from the camera to the object gets shorter.

(7) $$\mathrm{error}\left(\%\right)=\frac{\left||Th-Emp|\right|}{Emp}\times 100$$

The GSD empirical measurement let us also assess orientation deviations in the camera positioning that could potentially affect the spatial axis (*X*-axis). A correct alignment of the device is critical to avoid further distortions. Having all red and blue crosses equidistant suggests that the camera is aligned with respect to the linear stage and, therefore, black and white squares measure the same. In our case, a maximum deviation of one pixel has occurred between distances, so camera roll and yaw are considered to be zero.

Deviations in the camera pitch angle have an impact on the lambda axis (*Y*-axis) and are somehow much harder to measure empirically. Thus, in this case, a level has been used to check that the angle is very close to 0.

After having performed the aforementioned measurements and calculations, the final step consists of checking that the motor motion and the camera acquisition are perfectly synchronized. This is accomplished by performing a morphological analysis of an image of an object with a circular shape of 3 cm of radius, fitting an ellipse onto the shape and measuring the ratio between the shortest and longest axis. A ratio close to one indicates a proper synchronisation.

Being more specific, in case the GSD value used to scan the circular shape is accurate enough, the camera-captured lines contain no overlapped or missing information so the circular shape can be perfectly reconstructed in the image after stacking all lines together. When fitting an ellipse onto it, the major and minor axes would have the same length. However, if the GSD is not optimal, the acquired image lines would either overlap (if the GSD is larger than what it should be) or miss information (when the GSD is smaller than what it should be). The resulting image would then have an ellipsoidal shape which would result in its major and minor axes being of different length.

**Figure 11:**
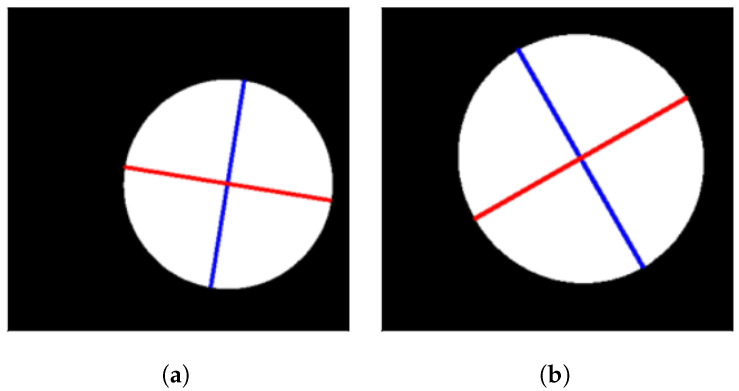
Morphological analysis of the proposed system using the Specim FX10 camera. (**a**) Binary image of the acquired circular object captured using the empirical GSD. Fitted ellipse axes are shown in red and blue. (**b**) Binary image of the acquired circular object captured using the theoretical GSD. Fitted ellipse axes are shown in red and blue.

Figure 11 shows the binarized result of two images acquired with the FX10 camera changing the pixel GSD value with the circular object place at a height of 920 mm. Figure 11a shows the acquired image with the empirical GSD, 0.641mm/px, which was calculated before and displayed in Table 4 and Figure 11b shows the acquired image with the theoretical GSD, 0.618mm/px, which was obtained using Equation (2).

The approach followed to obtain the ellipse axes is explained in [33]. First a principal component analysis (PCA) [34] is applied to the image to retain a single component, where the background and circular object are much easier to split. An algorithm from the Open Computer Vision (OpenCV) library [35] is applied to fit an ellipse to the previous result. Results for the two images displayed in Figure 11 can be seen in Table 5. Though both shapes look rather perfectly circular, looking at the numbers the empirical GSD value provides a ratio slightly closer to one than the theoretical value; therefore, a correction factor specific for each camera has been introduced in the software to account for the deviations in all hyperspectral image acquisitions carried out with the platform. This correction factor is calculated as the relation between the empirical GSD value and the theoretical GSD value. This factor corrects all the inaccuracies introduced in the equation such as the hand-measured working distance.

## 5. Use Case: Plastic Samples Acquisition

Around 300 million tons of plastic is produced worldwide every year, and over 8 million of that enters the oceans [36]. Many marine animals become tangled in plastic or consume it and as a result are dying en masse. On top of that, there are several studies that explore the relationship between human health problems and consuming fish that contain plastics [37]. At this point, no one denies that oceanic ecosystems around the world have been ravaged by plastic waste.

In order to diminish the impact of plastic in the world, recycling plays a key role; however, still more than 90% of plastic ends up simply in waste without being reprocessed into useful products. To this end, sorting the waste into the different type of plastics is essential. In recent years, plastic sorting using HSI techniques has gained momentum due to the improvements in the results as it uses additional information for the classification process rather than just the color, which is not particularly related to the material composition.

**Figure 12:**
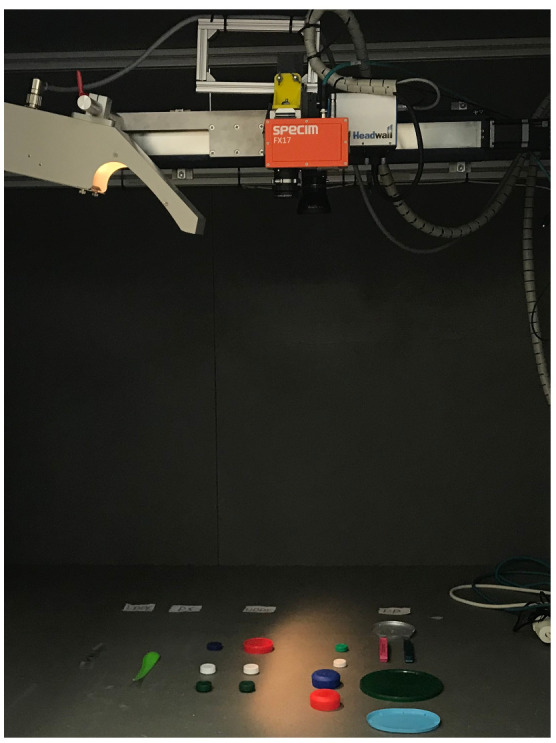
Plastic samples layout in the proposed hyperspectral acquisition system.

In this work, different types of plastics such as Polypropylene, Polystyrene and High-Density Polypropylene have been scanned using the proposed system. Figure 12 shows the hyperspectral system proposed in this work with the mentioned plastic samples at the bottom, ready to be scanned.

The developed Software GUI presented in Section 3 integrates an image-inspection tab as well, which allows a first inspection of the captured data, proving that the acquisition process has produced meaningful spectra.

**Figure 13:**
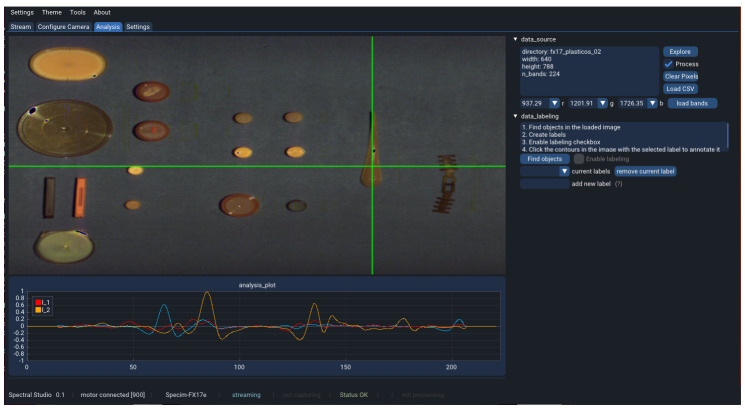
Plastic samples analysis using the developed software tool.

This data exploration is carried out in the *Analysis* tab which presents a layout similar to the *Stream* tab, with an image widget on the left hand side, a plot widget underneath it and some controls on the right hand side. Figure 13 displays the graphical tool after the plastic samples image has been loaded, once the user has selected a directory where the hyperspectral image and its corresponding ENVI header file are located.

The application displays the image in the widget using the default bands that are defined in the header file. As an additional feature, the user can select in the interface which wavelengths to use to display the image in case other bands than the default are to be explored.

Within the image widget boundaries, a mouse left-click plots the spectral signature of the selected pixel. This allows the user to easily obtain a visual representation of the spectral characteristics of the scanned objects. Right-clicking on top of the image will make the selected pixel spectral signature persistent in the plot so that the user can easily compare the spectral data of different pixels of the image. Furthermore, there are a few predefined options to process the spectral data before plotting it in case some specific applications require it. For instance, in the plot widget, the second derivative of the pixel spectrum of the individual plastic types is displayed after being normalized and smoothed. This processing step can be enabled and disabled at any time as well as the tuning of the processing parameters, in the *Settings* tab. Spectral data can also be imported from external comma-separated-value (CSV) files and plotted together with the pixel spectra. This allows the user to compare external data with the one captured by the proposed system.

**Figure 14:**
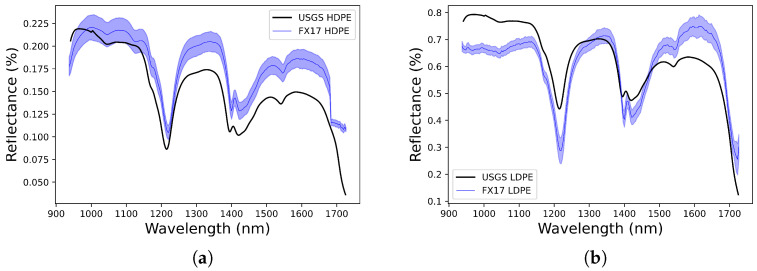
Comparison between the USGS plastics and the plastics captured by the Specim FX17. (**a**) HDPE plastic comparison. (**b**) LDPE plastic comparison.

One way to validate the spectral signatures obtained by the developed system is to compare them with existing spectral libraries, such as the United States Geological Survey (USGS) [38]. Figure 14 shows the spectral signature of two different plastic types along with the variability of each captured spectral signatures, HDPE shown in Figure 14a and LDPE shown in Figure 14b, in the spectral range 900 to 1700 nm, taken from the USGS Spectral Library and captured by the Specim FX17 camera using the developed system. Samples of 100 pixels have been selected to obtain the mean spectral signature value in order to compare it with the spectral signature of the corresponding plastic type from the external library.

In order to be able to numerically quantify the results, two metrics have been used. First, the spectral angle between the captured samples and the USGS references has been calculated. Spectral angles of $5.378^{\circ }$ for the HDPE plastic and $6.846^{\circ }$ for the LDPE plastic have been obtained. These values are very close to 0 which indicates a very high similarity degree.

Secondly, the spectral variability present in the measured data for a particular plastic type has been estimated by measuring the spectral angle standard deviation of the selected pixels against the external library reference. This provides an outcome per spectral band, which is then averaged in order to acquire a variability figure for the assessment. A mean variability of 0.00911 for the HDPE plastic and 0.02498 for the LDPE plastic have been obtained.

## 6. Conclusions

In this work a laboratory hyperspectral acquisition system has been engineered based on a linear displacement, an appropriate illumination system, a sealed cage to contain the light, mechanical 3D modelling and software modules supported by open-source packages. This permits the acquisition of images with any hyperspectral pushbroom camera following the GigEvision interface standard, which is gaining momentum due to its simplicity compared to other cameras. Moreover, a smart calibration procedure has been proposed which does not require the capturing of a white and dark samples every time an image acquisition takes place, as it keeps a record of all samples and applies the most suitable one. On top of that, the system has been validated in terms of checking whether aberrations are properly corrected within the device, the spectral response compared against a known reference and the synchronisation between motor and camera frame rate are fine-adjusted. This work introduces different methods for detecting and quantifying some of the most common spectral aberrations with very low-cost equipment. Finally, the solution has been used to acquire different plastic samples and show their spectral signatures. This entails a huge potential, for instance, in plastic inspection and sorting as well as creating a database of plastic signatures that can be used in segmentation and classification applications. As a future line of work, the aim is to create an online database to which not only our group, but any researcher can contribute their spectral data. For that purpose, it is also our intention to make the analysis software available to the community so researchers can utilise it for the indicated purpose.

Given that hyperspectral technologies are increasingly on the rise, it is our intention that this work can serve as a starter manual for any research group that wants to set up their own hyperspectral laboratory. The hyperspectral capturing booth presented in this work offers very useful features, especially for research purposes. We have explained both the details that must be taken into account to achieve a correct capture of spectral information and techniques that can be used to validate the correct operation of the system. First, the whole system is designed to work with a wide range of pushbroom cameras as the only requirement for them is to comply with the GenICam protocol. This gives the user a lot of flexibility, making it possible to use this setup for a wide range of spectral applications. Secondly, the controlled environment conditions is further profited by storing additional measured data of all spectral calibration references. This permits the reuse of those references when performing spectral captures in similar conditions, avoiding the repetitive task of capturing white and dark references prior to every capture. Thirdly, the fact that all software modules used in this development open source also grants a high flexibility while representing a lower-cost approach compared to market solutions. The techniques proposed in this work for detecting spectral aberrations are quite easily reproduced as they do not require additional spectral equipment. Nonetheless, they can not replace the expensive equipment used by camera manufacturers in their laboratories for quantifying spectral aberrations. As another future line of work, the intention of our research group is to acquired this laboratory equipment to keep improving the process of quantifying the aberrations.

## Figures and Tables

**Table 1:** Specim FX10 and FX17 main characteristics.

	FX10	FX17
Spectral Range	400–1000 nm	900–1700 nm
Spectral Bands	224	224
Spatial Sampling	1024 px	640 px
Spectral FWHM	5.5 nm	8 nm
Spectral Resolution	2.7 nm	3.5 nm
Frame Rate	327 FPS	527 FPS
FOV (α)	38°	38°
Camera SNR (Peak)	420:1	1000:1
Dimensions	150 × 85 × 71 mm	150 × 75 × 85 mm
Weight	1.26 kg	1.56 kg
Sensor Material	CMOS	InGaAs
Camera Interface	GigE	GigE
Effective slit width	42 μm	42 μm
Bit depth	12	12

**Table 2:** Zaber A-LST1000B linear stage main characteristics.

Maximum Centered Load	1000 N
Maximum Cantilever Load	3000 N·cm
Maximum Continuous Thrust	350 N
Microstep Size (Resolution)	0.4961 μm
Travel Range	1000 mm
Backlash	<10 μm
Maximum Speed	100 mm/s
Minimum Speed	0.000303 mm/s
Weight	6.90 kg
Communication Interface	RS-232
Communication Protocol	Zaber ASCII/Zaber Binary

**Table 3:** Keystone empirical results measured in pixels.

	Specim FX10		
	w. AIE	w/o. AIE	Correction (%)
left side	0.86597	4.55817	81.00
right side	0.07689	3.99435	98.07
center	0.04523	1.07568	95.79
	**Specim FX17**		
	**w. AIE**	**w/o. AIE**	**Correction (%)**
left side	0.94518	1.47291	35.82
right side	0.82366	1.38280	40.43
center	0.03998	1.12169	96.43

**Table 4:** GSD empirical measurements results.

	Specim FX10		
**H (mm)**	**GSD (mm/px)**		**Error (%)**
	**Theoretical**	**Empirical**	
280	0.188	0.216	12.96
680	0.457	0.476	3.99
920	0.618	0.641	3.58
	**Specim FX17**		
**H (mm)**	**GSD (mm/px)**		**Error (%)**
	**Theoretical**	**Empirical**	
280	0.301	0.325	7.38
680	0.731	0.778	6.04
920	0.989	1.014	2.45

**Table 5:** Morphological analysis results.

	Ellipse Axis Ratio	
	Fx10	Fx17
GSD theoretical (h = 920 mm) [mm/px]	0.9766	0.9701
GSD empirical value [mm/px]	0.9986	0.9814
